# Do irrigation solutions effect bond strength of composite resin to deep margin elevation material? An in-vitro study

**DOI:** 10.1186/s12903-025-06229-2

**Published:** 2025-05-28

**Authors:** Şeref Nur Mutlu, Yasemin Derya Fidancıoğlu, Hatice Büyüközer Özkan, Hayriye Esra Ülker

**Affiliations:** 1 Health Vocational School, Necmettin Erbakan University, Konya, 42200 Turkey; 2 Department of Endodontics, Faculty of Dentistry, Alanya Alaaddin Keykubat University, Alanya, Antalya Turkey; 3 Department of Operative Dentistry, Faculty of Dentistry, Selcuk University, Konya, Turkey

**Keywords:** Deep margin elevation, Irrigation solution, Flowable composite, Micro-tensile bond strength

## Abstract

**Background:**

The deep margin elevation (DME) technique provides advantages for root canal treatment, but the impact of applied irrigation protocols on bonding for subsequent restorations is a significant concern. The aim of this in vitro study was to test the bond strength of a flowable resin material used in DME restorations after composite restorations were exposed to different irrigation protocols.

**Methods:**

Standard composite samples (G-aenial^®^ Universal Injectable) were divided into nine groups according to irrigation protocols. Untreated composite sample were used as control. The group A were kept in 5.25% sodium hypochlorite (NaOCl) and the group B were kept in 3.5% chlorine dioxide (ClO_2_) for 30 min. Then, the following treatment protocols were applied to the subgroups, respectively. Group1A/B: 17%EDTA + 5.25%NaOCl + Distilled Water + 2%CHX, Group2A/B: 18%HEDP + 5.25%NaOCl + Distilled Water + 2%CHX, Group3A/B: 17%EDTA + 3.5%ClO_2_ + Distilled Water + 2%CHX, Group4A/B: 18%HEDP + 3.5%ClO_2_ + Distilled Water + 2%CHX. After irrigation procedures, samples were washed with distilled water and sandblasted. G-Premio Bond and composite restorations (G–aenial^®^ A’CHORD) were applied. The samples were then cut perpendicular to the interface with an IsoMet^®^ low speed diamond saw under water. For the microtensile bond strength test, rectangular sticks with an average cross-sectional area of ∼1 mm^2^ will be obtained. The first section with 1-mm thickness was excluded to prevent its possible confounding effect on the results. Three sections were obtained of each sample (*n* = 15) and tested for microtensile bond strength. The analysis of the data collected in accordance with the purpose of the study was performed with One-way ANOVA (*n* = 15). For multiple comparisons between groups, it was evaluated with Tukey HSD test.

**Results:**

Groups A1 and A2, immersed in NaOCl for 30 min, showed statistically significantly lower bond strength compared to Group B3, immersed in ClO₂ for 30 min, and the control group (*P* < 0.05). The use of ClO₂ irrigation is recommended due to its positive effects on bond strength.

**Conclusion:**

Prolonged NaOCl irrigation may adversely affect the bond strength of flowable composites used for deep margin elevation.

## Background

The primary goal of root canal therapy is to remove inflamed or necrotic pulpal tissue and reduce or eliminate the presence of microorganisms [1]. The anatomic complexity of the root canal system represents a challenge for mechanical instrumentation in effectively removing all contaminated tissues and bacteria present in isthmuses and ramifications. Several solutions, including sodium hypochlorite (NaOCl), chlorine dioxide (ClO_2_), ethylenediaminetetraacetic acid (EDTA), etidronic acid (HEDP), and chlorhexidine (CHX), have been proposed and used as irrigants in various studies [2–5]. A combination of NaOCl and EDTA has been shown in many experimental studies to be highly successful in the elimination of inorganic and organic tissues [6]. HEDP, which is also referred to as etidronic acid or etidronate, is used as a systemic treatment for osteoporosis and Paget’s disease [7]. It has been proposed as an alternative for smear layer removal instead of EDTA, since etidronic acid (HEDP-HEBP) may be combined with NaOCl without changing its antibacterial or dissolutive properties, making it a promising alternative [8].

Chlorine dioxide (ClO₂) is a versatile chemical agent known for its biocompatibility, strong oxidative properties, and wide spectrum of antimicrobial activity. Its ability to disrupt bacterial cell walls and denature proteins makes it a promising candidate for disinfection procedures in dentistry, including endodontics. Unlike NaOCl, which is traditionally used in root canal irrigation, ClO₂ exhibits effective antimicrobial properties with potentially less cytotoxicity to periapical tissues [9, 10]. Büyüközer et al. [11] investigated the chemical interactions of ClO₂ with various irrigants and demonstrated that its application in root canal systems, when activated by photon-induced photoacoustic streaming, significantly improved canal cleanliness without compromising dentin structure. Similarly, Herczegh et al. [12] reported that a high-purity ClO₂ solution effectively eradicated Enterococcus faecalis biofilms within the root canal, which are often resistant to conventional irrigants. These findings highlight ClO₂’s potential as an alternative to NaOCl in endodontic therapy, offering effective disinfection with a favorable safety profile.

The presence of limited residual dental tissue represents a considerable challenge, which makes endodontic procedures particularly complex. The application of pre-endodontic restoration prior to endodontic treatment is considered advantageous for teeth that exhibit weakness [13]. According to Tanikonda [13], pre-endodontic restoration plays a crucial role in achieving optimal isolation of the rubber dam during subsequent endodontic visits. Pre-endodontic treatments help facilitate post-endodontic restoration. With the development of adhesive systems, the use of traditional non-adhesive pre-endodontic restoration techniques such as amalgam cores, copper bands or temporary crowns, which have many limitations, has decreased [14, 15]. Deep margin elevation (DME) technique which is more current today, provides space for the long-term operation of irrigation solutions, thereby enhancing their effectiveness [16]. According to Naum & Chandler [17], emphasized that short- and long-term restorations applied during and immediately after endodontic treatment are important factors for preventing bacterial microleakage, avoiding the seepage of medicaments into the dental pulp, and preserving pulp vitality as well as ensuring the success of regenerative endodontic procedures. Additionally, ElAyouti et al. [18], treatment that prevents microbial contamination helps to avoid the rupture of the weakened tooth structure, thereby preserving its structural strength.

The DME technique aims to restore the approximal dentin position by applying a composite resin base onto the pre-existing cervical margin, thus elevating the border coronally [16, 19]. Following the application of a composite resin base to the cervical margin, which can enhance the success of canal treatment procedures in teeth with excessive loss of substance, bonding with direct restorative composite resin after various irrigation protocols used during canal treatment becomes crucial. While this step-up technique provides advantages for canal treatment, the impact of the applied irrigation protocols on bonding for subsequent restoration is an important concern. While numerous studies have been conducted on dental bonding, there is limited research on materials used in elevated steps.

Teeth requiring root canal treatment rarely have intact crowns and may have old, leaking restorations that need to be replaced or removed [20]. The restoration applied after endodontic treatment is as important as the procedures performed during the endodontic treatment process for clinical success. Problems are encountered in the restoration of deep proximal caries with subgingival margins after endodontic treatment. DME technique is to elevate proximal dentin coronally by applying a composite resin base on the cervical margin. However, in this case, the materials used for DME are exposed to the irrigation solutions applied during root canal treatment.

The aim of this in vitro study was to evaluate the effect of various irrigation solutions on bond strength of a flowable resin material used for DME to composite resin. The null hypothesis was that there would be no significant change in the bond strength of the flowable resin material used in DME restorations to composite restorations after exposure to different irrigation protocols.

## Materials and methods

The study was approved by the Necmettin Erbakan University Faculty of Dentistry Ethics Committee for Non-Drug and Medical Device Research (Konya, Turkey) (2022/211- 11707).

Statistical Analysis According to the power analysis results for the Bivariate Normal Model performed with the G Power program (G * Power 3.1 software; Heinrich Heine University, Düsseldorf, Germany), with α (error margin) = 0.05, at a power of 0.90 (1-β) critical correlation value of 0.3, 10 samples were determined to be suitable.

**Fig. 1:**
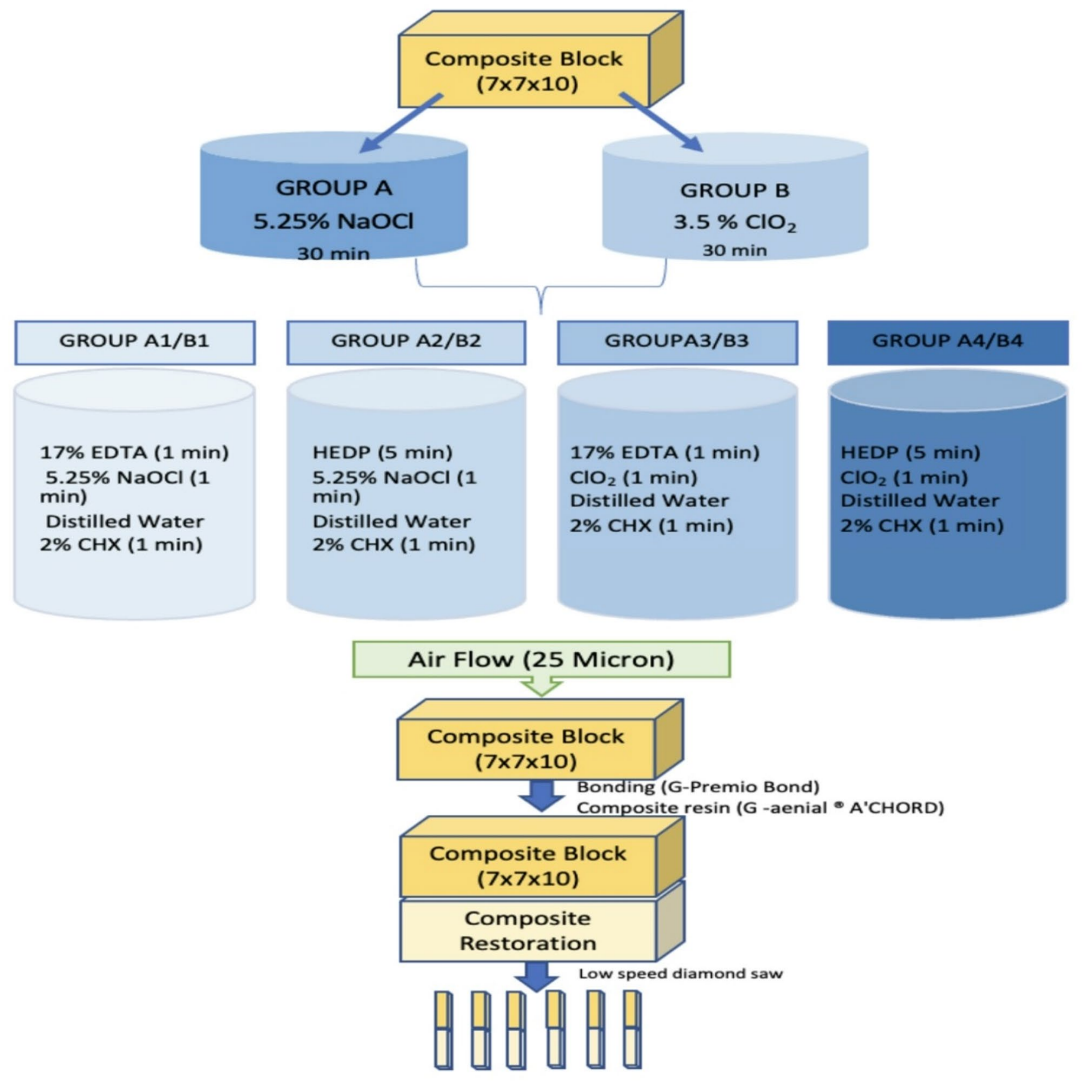
Schematization of the experimental groups and the procedure

Forty-five composite (G-aenial^®^ Injectable GC corporation, Tokyo, Japan) resin blocks were made by silicone mold (7 mm x 7 mm x10 mm). Composite was applied into the silicone mold in 2 mm increments using a titanium tipped tool and condensed completely. The samples were light-cured for 20 s at a distance of 1 mm using a LED light-curing unit (Woodpecker LED.F, China) with a light intensity of 1000 mW/cm^2^. The samples were removed from the mold and then the surface of the composite blocks was polished with a 600-grit abrasive to create a uniform surface. Composite samples were then divided into two and then four subgroups according to irrigation protocols. Composite blocks divided into two groups were immersed in group A to 5 ml 5.25% NaOCl and in group B to 5 ml 3.5% ClO_2_ for 30 min to simulate irrigation during root canal preparation. The solutions were renewed every 10 min [11, 21]. These two groups were then divided into four subgroups and different final irrigation protocols were applied to the samples. For each sample, 5 ml of fresh solution was applied for the times indicated in Fig. 1. The composite block in the control group received no irrigation.

Irrigation solutions were applied to the composite blocks according to the following protocols.

Group A1; 5.25% NaOCl (30 min) + 17% EDTA (1 min) + 5.25% NaOCl (1 min) + Distilled Water (1 min) + 2% CHX (1 min),

Group A2; 5.25% NaOCl (30 min) + 18% HEDP (5 min) + 5.25% NaOCl (1 min) + Distilled Water (1 min) + 2% CHX (1 min),

Group A3; 5.25% NaOCl (30 min) + 17% EDTA (1 min) + 3.5% ClO_2_ (1 min) + Distilled Water (1 min) + 2% CHX (1 min),

Group A4; 5.25% NaOCl (30 min) + 18% HEDP (5 min) + 3.5% ClO_2_ (1 min) + Distilled Water (1 min) + 2% CHX (1 min),

Group B1; 3.5% ClO_2_ (30 min) + 17% EDTA (1 min) + 5.25% NaOCl (1 min) + Distilled Water (1 min) + 2% CHX (1 min),

Group B2; 3.5% ClO_2_ (30 min) + 18% HEDP (5 min) + 5.25% NaOCl + Distilled Water (1 min) + 2% CHX (1 min),

Group B3; 3.5% ClO_2_ (30 min) + 17% EDTA (1 min) + 3.5% ClO_2_ (1 min) + Distilled Water (1 min) + 2% CHX (1 min),

Group B4; 3.5% ClO_2_ (30 min) + 18% HEDP (5 min) + 3.5% ClO_2_(1 min) + Distilled Water (1 min) + 2% CHX (1 min).

After the Irrigation process was completed, The composite blocks air abraded (AIRFLOW^®^ Prophylaxis Master, EMS, Geneva, Switzerland) with 40-micron Sodium Bicarbonate powder (Air Flow Classic, EMS, Geneva, Switzerland). Sandblasting application provides higher bond strength by creating a micro-retentive surface [22]. The sandblasting procedure was performed for 10 s at a constant distance (5 mm) and angle (30°) from the irrigated surface in all groups, as described.

G-Premio Bond (GC Corporation, Tokyo, Japan) was applied to composite blocks for 10 s, followed by 5 s of gentle air spray and light cured for 10 s. The composite resin (G -aenial^®^ A’CHORD, GC Corporation, Tokyo, Japan) was then incrementally applied condensed wholly and light-cured (20 s) to the prepared samples (Table 1).

The composite blocks were then glued to acrylic blocks and cut perpendicular to the interface with an IsoMet^®^ low speed diamond saw (Isomet, Buehler, IL, USA) with water coolant. For the micro-tensile bond strength test, rectangular sticks with an average cross-sectional area of ∼1 mm^2^ were obtained. The first section with 1-mm thickness was excluded to prevent its possible confounding effect on the results. Three sections were obtained of each sample (*n* = 15). The cross-sectional area of each sample was measured with a digital calliper (Mitutoyo, Tokyo, Japan). Each sample was fixed with cyanoacrylate gel and the adhesive interface remained free. The samples were then fixed to the universal testing device (TSD 500, Chatillon-Ametek, Agawam, MA, ABD) in a line with the direction of the applied tensile force, which was done so at a crosshead speed of 1.0 mm/min. Data was converted to MPa before statistical analysis and the micro-tensile bond strength was calculated.

**Table 1:** Test materials, manufacturers, lot numbers and compositions

Materials	Compositions
G-Premio BOND GC Corporation, Tokyo, Japan 1,907,221	Acetone (25–50%), 2-hydroxy-1,3-dimethacrylaxypropane (10–20%), methacryloyloxydecyl dihydrogen phosphate (5–10%), 2,2-ethylenedioxydiethyl dimethacrylate (1–5%), diphenyl(2,4,6-trimethylbenzoyl) phosphine oxide (1–5%), 2,6-di-tert-butyl-p-cresol (< 0.5%).
G-aenial Universal InjectableGC Corporation, Tokyo, Japan 2,204,071	Dimethacrylate, Barium (Ba) glass, Silicon dioxide (SiO_2_)
G-aenial Achord GC Corporation, Tokyo, Japan 2,204,081	Bis-MEPP, filler load: 82% by weight: Glass-filler (300 nm barium glass) 16 nm (fumed silica), organic filler (300 nm barium glass; 16 nm fumed silica).

### Statistical analysis

Difference between groups were performed with one-way analysis of variance (ANOVA) after the normal distribution test (Shapiro-Wilk and kurtosis/skewness) using the Statistical Package for the Social Sciences (SPSS) version 22.0 (SPSS Inc, Chicago, IL, USA) statistical package program and evaluate it with the Tukey HSD test for multiple comparisons between groups.

## Results

The bond strength values for different irrigation protocol groups are summarized in Table 2. Statistically One-way ANOVA compared different irrigation protocols and control group and revealed significant differences among the groups at *P* = 0.003 (Table 2).

The bond strength of control group was 37.44 MPa, Group A, which was kept at 5.25% NaOCl for 30 min, were 25.26, 25.12, 29.69 and 29.60 MPa, respectively. The bond strengths of Group B, which were kept in ClO_2_ for 30 min, were 34.55, 29.51, 36.81 and 31.94 MPa, respectively (Table 2).

Group A1 and Group A2, kept in NaOCl for 30 min, showed statistically lower bond strength than the Group B3, kept in ClO_2_ for 30 min, and control group (*p* < 0.05). All other groups showed similar bond strengths to control group and each other (*p* > 0.05).

**Table 2:** Average bond strength (MPa) of the groups, P reveals statistical differences from the control group (*p* = 0.05)

Groups	Subgroups	*N*	Mean (MPa)	*p*
Control	Control	15	37.44 ± 4.38^a^	
**Group A** **NaOCl** **(30 min)**	Group A1 EDTA + NaOCl + DW + CHX	15	25.26 ± 10.15^b^	0.005
	Group A2 HEDP + NaOCl + DW + CHX	15	25.12 ± 6.10^b^	0.004
	Grup A3 EDTA + ClO_2_ + DW + CHX	15	29.69 ± 7.59^ab^	0.252
	Grup A4 HEDP + ClO_2_ + DW + CHX	15	29.60 ± 11.87^ab^	0.238
**Group B** **ClO**_**2**_ **(30 min)**	Grup B1 EDTA + NaOCl + DW + CHX	15	34.55 ± 6.72^ab^	0.991
	Grup B2 HEDP + NaOCl + DW + CHX	15	29.51 ± 7.46^ab^	0.225
	Grup B3 EDTA + ClO_2_ + DW + CHX	15	36.81 ± 10.14^a^	1.000
	Grup B4 HEDP + ClO_2_ + DW + CHX	15	31.94 ± 9.86^ab^	0.708

The difference between the means carrying different letters on the same column is significant.

## Discussion

In this study, the microtensile test was chosen for examination to determine whether different irrigation procedures had an effect on the bonding of the DME material with the composite filling material. The findings showed that the bond strength decreased in the groups subjected to NaOCl in final irrigation following 30 min NaOCl irrigation. In irrigation protocols combined with ClO_2_, bond strength did not decrease even with 30 min of NaOCl pretreatment. In this case, the working hypothesis was rejected. When the bonding strength of these composite surfaces exposed to irrigation solutions were evaluated, the most successful group was group B3, which was applied EDTA, ClO_2_, DW, and CHX after 30 min of ClO_2_ application, respectively, and showed similar results with the control group.

DME technique, introduced by Dietschi and Spreafico [19], is also referred to as “cervical margin relocation,” “proximal box elevation,” and “coronal margin displacement,” and offers several advantages in terms of isolation [16]. Magne et al. [16], recommend performing DME before endodontic treatment to benefit from improved isolation during root canal treatment.

Regarding the best dental material for DME, there is no agreement. Dental professionals can use any of these materials to restore deep proximal boxes because DME restorations with appropriate marginal adaptation can be made with bulk fill resin-based composites, conventional resin-based composites, flowable resin-based composites, and glass ionomer cement or resin-modified glass ionomer cement [23, 24]. Flowable composites outperform bulk-fill and nanohybrid composites in terms of marginal sealing, as shown by Scotti et al. [25].The highly filled G-aenial Universal injectable flowable composite resin was used in our investigation. For all restorative indications, G-aenial Universal is an injectable, light-curing, radiopaque, universal, high-strength composite with low viscosity. Highly-filled flowable resin composite demonstrated promising results when used as a liner, as demonstrated by Baldi et al. [26].

Dietschi et al. [27] observed that using a material with a moderate elastic modulus, such as flowable composites, results in superior internal adaptation compared to harder materials. A flowable composite serves as a stress-absorbing layer beneath the hybrid composite resin restoration. This concept is supported by the notion of an “elastic wall” characterized by a low elastic modulus and high wettability, where the incorporation of flowable materials functions as an intermediary layer. This intermediary layer not only mitigates the stress caused by polymerization shrinkage but also alleviates stress during functional loading. Increasing the thickness of this layer enhances its stress-absorbing capacity. The efficacy of stress absorption by this layer is contingent upon its thickness and modulus. Conversely, a separate study revealed no significant variance in marginal fit among different composite types [27–29]. In the current study, the idea of elevation the margin before root canal treatment is based on several reasons, the first of which is to eliminate the problem of not being able to seal the margin of the tooth by the temporary filling in the intermediate stages of root canal treatment, the second is to create a pool in the pulp cavity and ensure better activation of irrigants in the canals during root canal treatment, and the third is to ensure the success of the final restoration.

There are many studies in the literature on the effects of irrigation solutions on dentin and their effects on bonding with composite fillings [30–32], but no studies have been found on their effects on restorative materials. Several studies have been found regarding the effect of NaOCl irrigation on degradation of the resin-dentin interface. In the study of Yamauti et al. [33], bond strengths decreased with increasing storage time in NaOCl and NaOCl was responsible for the deterioration of the bonds on the hybrid layer. Unlike the previous study, Sacramento et al. [34], concluded in their study that NaOCl was not harmful to dentin adhesion and that microtensile bond strength test values did not decrease in groups irrigated with NaOCl after 90 days of storage in water. Furthermore, NaOCl decomposes into oxygen and sodium chloride, which can disrupt the polymerization of resin sealers, resulting in a significant inhibition at the resin/dentin interface and a weaker bond [35]. While in these studies, NaOCl was applied to dentin surfaces, in our study, unlike these studies, NaOCl was applied to the surface to composite and it was observed that the connection was negatively affected.

In a study comparing the effects of chlorine dioxide and sodium hypochlorite on the dissolution of human pulp tissue, 2% NaOCl, 5% ClO_2_, and isotonic saline solution (control) were used [36]. The results showed that NaOCl dissolved human pulp tissue more effectively than ClO_2_. Considering the limitations of this study, it can be concluded that 5% ClO_2_ can dissolve human pulp tissue but is less effective than 2% NaOCl [36]. Furthermore, there are studies indicating that changing the concentration of chlorine dioxide can enhance its ability to dissolve tissue. According to the results, when used at a low concentration (0.12%), hyper-pure chlorine dioxide (hClO_2_) showed similar effectiveness as NaOCl. Both disinfectants have partial ability to eliminate bacteria in dentin tubules. Therefore, hClO_2_ can be used at a lower concentration while achieving the same efficacy [37]. Considering all these, it may be preferable to use chlorine dioxide as an irrigation solution. In our study, NaOCl and ClO_2_, were preferred and it was found that the effect of ClO_2_ on the bond between the restorative material and the flowable composite was lower compared to NaOCl and closer to the control group. In previous studies, it has been reported that solutions commonly used in clinics such as NaOCl, EDTA, CHX, or normal saline can affect the chemical properties of restorative materials and the bond strength to dentin [30–32]. Additionally, exposure to NaOCl significantly reduced the bond strength of NeoPutty and Well-Root PT, which is used as a perforation repair material [38]. In our study as well, all groups treated with NaOCl had lower bond strength compared to the control group. This is believed to be due to NaOCl dissolving the organic content of the material and reducing surface fracture resistance.

Different chelating agents have shown varying potentials to limit the impact of NaOCl [39–41]. The chelating agent that is most frequently employed is EDTA [42, 43]. However, when NaOCl and EDTA are used combined, demineralization and peritubular and intertubular dentin erosion have been noted [44, 45]. Furthermore, a procedure that alternates between using EDTA and NaOCl changes the NaOCl capacity to dissolve organic materials [46, 47].

To solve the above mentioned problem, a distinct method called continuous chelation was introduced in 2005. This approach suggested using NaOCl with weak chelating agents that consume less free available chlorine than EDTA and other strong chelators The potential use of HEDP for this purpose is reported [8]. HEDP is one kind of non-nitrogenous bisphosphonate [48]. Etidronate, a salt with cations attached to the HEDP anion (often Na2HEDP or Na4HEDP), is another form of it [49]. It should not be used as a single irrigation since it is a weak chelating agent. To get best impacts, it should be applied as 300 s [50]. In addition to its low toxicity, it prevents the formation of the smear layer when used in continuous chelation during biomechanical preparation [51]. In our study, HEDP was preferred and applied due to these advantages. However, it can be said that the use of HEDP or EDTA did not make a difference between the groups in the results of the study. There is still no consensus in the literature on the corrosive potential of EDTA and HEDP. Previous studies stated that irrigation with a NaOCl/HEDP mixture was no different from sequential irrigation with 3% NaOCl and EDTA [52, 53].

Due to its superior antibacterial ability and important properties, CHX is the most commonly used cavity disinfectant and root canal irrigation solution [4]. The use of CHX as the final irrigation solution has been found to enhance disinfection of the root canal system but has no weakening effect on bond strength. In our study, CHX was preferred as the final irrigation [54].

However, although there is no study on the bond strength of CHX on composite surfaces, it is thought that it has no effect on the bond in our study. In fact, the meta-analysis conducted by Zhang et al. [55] stated that the use of 0.1% and 0.2% CHX did not negatively affect the immediate bond strength. In these in vitro clinical studies, it was stated that CHX maintained the stability of the bond strength when added to self-etching.

A direct correlation has been established between restoration quality and the clinical success of teeth that have undergone endodontic treatment. Without adequate restorative treatment, many endodontically treated teeth are lost. Composites and adhesive systems are widely used in various fields due to their aesthetic properties, ability to bond to enamel and dentin, and potential to enhance the integrity of the tooth-restoration complex.

The scope of this study was limited to comparing the effects of the tested irrigants on the surface of the flowable composite with their effects on the bond strength to the composite material. Different results may be found by activating the irrigating solutions or testing different restoration materials.

## Conclusion

When the flowable composite used as DME material is exposed to NaOCl during root canal treatment, its bond strength decreases significantly. Therefore, appropriate measures should be taken to ensure long-term restoration stability in case of NaOCl use. On the other hand, the combination of ClO₂ and EDTA showed the highest bond strength. Accordingly, preferring ClO₂ based irrigation protocols instead of NaOCl may provide advantages in preserving the bond strength of DME material. New studies to increase the bond strength of composite materials used for DME will contribute to this issue.

## Data Availability

All data sets used and/or generated in this work are obtainable from the corresponding author upon reasonable request.

## Abbreviations

DME: Deep margin elevation
NaOCl: Sodium hypochlorite
ClO_2_: Chlorine dioxide
EDTA: Ethylenediaminetetraacetic acid
HEDP: Etidronic acid
HEBP: Etidronic acid
SPSS: Statistical Package for the Social Sciences
ANOVA: One-way analysis of variance
hClO_2_: Hyper-pure chlorine dioxide
